# Increased Death-Rate in Nervous Disease

**Published:** 1919-12-06

**Authors:** 


### INCREASED DEATH-RATE IN NERVOUS DISEASE.

### A Result of the Past Hostilities.

No one who has carefully studied the many processes of metabolism involved in the efficient working of the human machinery, can fail to be struck by the intimate relationship and interdependence that exists between the different systems therein. It is absolutely impossible for one set of organs, comprising a system, to be put out of healthy action without giving rise to changes, often of a serious nature, in one, or all, of the other systems; thus, to take a familiar instance, what part of the human body is not affected by physiological and pathological changes in the circulatory system? For upon the condition of the blood depends the very existence of man; it might aptly be described as the sheet anchor to which he clings when attacked by his many formidable enemies.

### A Controlling Power.

Again, on the excretory organs the body is entirely dependent for the necessary elimination of toxic waste products which, if not removed, will rapidly bring about a fatal result. Over all these systems there is a controlling power, viz., the central nervous system; this dominates every thought, movement, and elaboration of which the body is capable; pitiable indeed, then, is the plight of the individual who is unfortunate enough to incur pathological changes at headquarters.

In the light of modern research it has been found that the nerves maintain to all parts a much more complicated service of transport than was at one time believed; thus there are many different kinds of nerve fibres, each of which set has its own allotted functions, in that it conveys specific impulses from the central nervous system to its destination, so that we have motor nerves carrying to the voluntary muscular system messages of action, causing those muscles to perform their own particular movements. Again, the afferent nerves carry sensations from the periphery and internal organs to their receiving-offices, which, when interpreted by the central nervous system, cause expressions of pain, heat, cold, etc.

In this article, however, we must confine ourselves to two sets of nerve fibres, viz. : (1) Those fibres which are concerned with the despatch of trophic, or nutritive, impulses. Without going into detail we can illustrate the action of these by considering the origin of bed-sores occurring in a patient who is suffering from nerve-paralysis, due to certain lesions in the central nervous system. (2) There is a set of nerves which subserves the function of carrying vaso-motor impulses, these latter being responsible for the maintenance of tone, etc.

Briefly to summarise the relationship between the tissues and their enemies, let us say, in popular phraseology, that the successful defence of the former against the latter depends upon sufficient powers of resistance. As with an army of men fighting a strenuous battle their success must very largely depend not only on the individual bravery and skill of the rank and file, but also on the orders issued to the latter by an intelligent Staff, who are acting upon inside knowledge impossible to the men under them, so also can the organs in the human body only keep themselves secure from the machinations of their invisible and ever active foes by the controlling influence of the headquarters Staff, viz., the brain, spinal cord, and accessory nerve-centres. Thus we see that the responsibility for the efficient co-operation of all classes dwelling within the body rests primarily upon the central nervous system, which, if itself should become the seat of disease, must cause demoralisation of all its subordinates.

### The Nation's Vitality.

The vitality of most people in the countries that have but lately emerged from the most devastating war in the history of the world must have been considerably impaired during the period of hostilities, and for some time afterwards. Actual starvation in many districts, great shortage of food in still more extensive areas, and everywhere the constant and ever increasing mental and physical strain, even in places far removed from the theatres of war, and warlike preparations, have undoubtedly precipitated a large proportion of persons into the border-lands between sanity and insanity; a region where psychoneuroses and other allied conditions hold sway.

Now, anyone who has treated cases of neurasthenia knows how greatly are diminished the powers of resistance in every department of the body; the terrible ravages of influenza showed this fact only too plainly. But when, superadded to the above war conditions, there are already present lesions affecting the central nervous system, must not the evil results of the former be markedly exaggerated?

